# A New Adaptive Synergetic Control Design for Single Link Robot Arm Actuated by Pneumatic Muscles

**DOI:** 10.3390/e22070723

**Published:** 2020-06-30

**Authors:** Amjad J. Humaidi, Ibraheem Kasim Ibraheem, Ahmad Taher Azar, Musaab E. Sadiq

**Affiliations:** 1 Control and Systems Engineering Department, University of Technology, Baghdad 10001, Iraq; 601116@uotechnology.edu.iq; 2 Department of Electrical Engineering, College of Engineering, University of Baghdad, Baghdad 10001, Iraq; ibraheemki@coeng.uobaghdad.edu.iq; 3 Robotics and Internet-of-Things Lab (RIOTU), Prince Sultan University, Riyadh 11586, Saudi Arabia; 4 Faculty of Computers and Artificial Intelligence, Benha University, Benha 13518, Egypt; 5 Ministry of Trade, General Company for Grain Processing, Baghdad 10001, Iraq; mesmsi50@gmail.com

**Keywords:** Pneumatic Artificial Muscles, synergetic control, adaptive control, particle swarming optimization, single-link robot arm

## Abstract

This paper suggests a new control design based on the concept of Synergetic Control theory for controlling a one-link robot arm actuated by Pneumatic artificial muscles (PAMs) in opposing bicep/tricep positions. The synergetic control design is first established based on known system parameters. However, in real PAM-actuated systems, the uncertainties are inherited features in their parameters and hence an adaptive synergetic control algorithm is proposed and synthesized for a PAM-actuated robot arm subjected to perturbation in its parameters. The adaptive synergetic laws are developed to estimate the uncertainties and to guarantee the asymptotic stability of the adaptive synergetic controlled PAM-actuated system. The work has also presented an improvement in the performance of proposed synergetic controllers (classical and adaptive) by applying a modern optimization technique based on Particle Swarm Optimization (PSO) to tune their design parameters towards optimal dynamic performance. The effectiveness of the proposed classical and adaptive synergetic controllers has been verified via computer simulation and it has been shown that the adaptive controller could cope with uncertainties and keep the controlled system stable. The proposed optimal Adaptive Synergetic Controller (ASC) has been validated with a previous adaptive controller with the same robot structure and actuation, and it has been shown that the optimal ASC outperforms its opponent in terms of tracking speed and error.

## 1. Introduction

Pneumatic actuators such as cylinders, pneumatic stepper motors, bellows, and pneumatic engines are commonly used to date. Pneumatic Artificial Muscles (PAMs) are one type of pneumatic actuator, which are made mainly of inflatable and flexible membrane that works like inverse bellows; i.e., they contract on inflation. The force generated by PAM actuators does not depend only on pressure, but also on the state of inflation, which adds another source of spring-like behavior. These PAMs, which mimic the animal muscle, are characterized by their light weight, since the membrane forms the core element of these actuators. However, they can transfer the same amount of power as cylinders do, when both actuators have the same volume and pressure ranges [1,2].

PAMs are used in many applications due to their light weight, simple construction and high force/weight ratio, direct connection, easy replacement and safe operation. The PAM actuators found their application in biomechanics, bio-robotics, robotics, artificial limb replacement Moreover, since PAMs are noise-free devices, they are applicable in hospital treatments to patients who are sensitive to noise. Compared to motor actuators, PAMs do not need a gear mechanism to increase power due to their high power/volume ratios. Due to their elasticity, PAMs are useful for the natural frequency of biped locomotion. Additionally, PAMs are useful for under-water applications due to their water immunity [3,4]. Since the operation of PAM mimicks that of real muscle, PAM is effectively used to implement the humanoid.

PAMs are characterized by many disadvantages. As compared to other actuators, one problem which can be addressed in PAMs is their antagonistic structure. This structure does not permit one PAM in the pair to contract and relax unless another PAM inflation causes the first PAM to deflate. However, only one motor is sufficient to perform the desired movement in traditional actuators [5]. One other main problem in PAMs is the difficulty to control them, since they are highly time varying and nonlinear and they involve uncertain parameters. This is due to the presence of various uncertain, nonlinear, and unknown terms in the system dynamics that restricts designing an effective tracking controller for the actuator. In addition, their high sensitivity to the working conditions of PAM systems such as viscosity, temperature and supplied pressure has greatly limited their working regions [6,7]. Many researchers have presented different control strategies to address the control problems of uncertain mechanical systems actuated by pneumatic muscles. The following literature addresses the recent control strategies applied to PAM-actuated systems:

Scaff et al., in [8], proposed an optimal conventional Proportional-Integral-Derivative (PID) controller for position control of one degree of freedom (DOF) system actuated by McKibben PAM. The terms of the PID controller are tuned using Simulated Optimization Algorithm (SOA), so as to obtain better dynamic performance of the PID-controlled system. One critical control problem is that the optimization is achieved off-line and there is no online adaption or optimization against variation of system parameters. Moreover, the PAM-actuated system is merely a hanging mass actuated by PAMs.

Choi et al., in [5], proposed a control technique for a PAM-actuated robot to replace a proportional pressure regulator (PPR) by a controller composed of a set of small encoders and pressure switches to solve the space problem due to the implementation of PPR. The results based on experimental tests show that the new controller could save space, but at the price of precision degradation. It is evident that the controller is based on an on-off technique and it did not address the uncertainty in system parameters, but it is intended to only save space.

Wang et al., in [9], proposed an adaptive fuzzy backstepping controller for motion control of a two-link anthropomorphic arm, which is subjected to frictions and model uncertainties. The PAM-actuated manipulator has been applied in upper-limb rehabilitation training. However, the high computation time, high memory storage and complex procedure of rule modification have been reported as the main drawbacks of using fuzzy adaptive controllers. In addition, the approximation in synthesizing a function-based model to represent the input–output mapping is another issue which degrades the performance of the proposed controller

Al-Jodah and Khames, in [10], designed a first and second order sliding mode control (SMC) for angular position control tracking of a single link robotic arm actuated by a pair of PAMs. A performance comparison has been made between the proposed controllers in terms of robustness against uncertainty in system parameters and in terms of their capability to suppress the chattering in their corresponding control signals. However, the work focused mainly on the solution of the chattering problem that is frequently attached to SMC design.

Medrano-Cerda et al., in [11], have designed an adaptive controller for the bi-muscular Pneumatic Muscle Actuator System. The control of the PAM system has been developed based on adaptive pole-placement control. In spite of the fact that the controller could give feasible accuracy and results in high power/weight ratio, the proposed adaptive scheme is synthesized based on indirect control methods, in which the mathematical model and system parameters is estimated according to online input–output acquired data based on proposed model structure.

Lilly and Yang, in [12], applied the sliding mode techniques for angle tracking of planar PAM-actuated manipulators, which are arranged in an agonist/antagonist set-up, under load exertion. The sliding mode controller is developed for the planar elbow manipulator so as to guarantee accuracy in the presence of modeling errors. The work did not address the adaptation in the controller to compensate the variation in system parameters. In addition, the presence of chattering behavior is an inevitable in control signal due to the inclusion of the hard switching function in the design of SMC methodology.

Jahanabadi, in [13], investigated the design of an integrated controller based on Active Force Control and FL (AFCFL) for position control of a PAM-actuated two planar link manipulator. The fuzzy logic is devoted to estimate the best value of the inertia matrix of the robot arm necessary for the AFC mechanism, which has been supported by a PID controller at the outer loop. Unfortunately, the work has applied a PID controller as the main tracking controller and the dynamic model has been approximated by a static gain, which is far from representing the actual model.

Tarapong and Radom, in [14], have designed an adaptive controller for tracking control of a one-link PAM-actuated robot arm under unknown physical system parameters. The work proved that the actual joint angle trajectory of an adaptive controlled system can track the desired trajectory within the prescribed tracking error in a finite time. The conducted results showed the robustness of the proposed controller under changes in robot parameters. However, the adaptive controller has been designed based on output tracking control with a different robot structure from the present work.

Repperger et al., in [15], presented a design of a gain-scheduled controller for regulating the response of a large-scale pneumatic muscle actuator. The scheduled gains are determined via many tests of transient and steady state responses. The design was primitive and has not taken into account the compensation due to variation in parameters of the PAM actuator.

Lilly, in [16], presented an adaptive technique for control tracking of joint angles of PAM-actuated limbs. The work conducted a comparison study between the proposed adaptive and PID controller. The results based on simulation showed the superiority of the proposed controller to the conventional one. However, the work has not addressed the design of an adaptive controller with both bicep and tricep PAMs attached to the arm, but there was an individual adaptive control design for each configuration; bicep or tricep PAMs attached to the arm.

Boudoua et al., in [17], proposed a neural network-based twisting sliding mode controller for control of a PAM-actuated robot arm and for chattering reduction in the control signal. The work used a two layer NN together with online adaptive learning law to approximate the unmodeled and unknown robot dynamics. This study lacked the ability to eliminate the chattering in the control signal and the use of NN structure for approximation could degrade the performance of the controller unless a suitable number and type of activation function is used.

Robinson et al. [18] studied the performance of three nonlinear control schemes, represented by sliding mode control, adaptive sliding mode control, and adaptive neural network (ANN) control for tracking control of a planar PAM-actuated manipulator. It has been shown that ANN controller is more robust against the variation in characteristics of the PAM actuator. The work concentrated on which controller needs the knowledge of the model more than the other. It has been concluded that the ANN controller is preferable since it does not need a model of the pneumatic system. However, the manipulator structure consists of a parallel PAM group that pulls a sliding plate, which rotates the link arm via tendon link about the pinned joint attached to the rigid body.

Shen [19] developed a sliding mode controller to cope with the uncertainties and disturbances of servo systems actuated by PAMs. The dynamic model of controllable canonical form was firstly developed based on flow, force, and pressure and load dynamics. One can report the appearance of chattering in the actuating signals due to the necessity of using the discontinuous function in design methodology.

Hosovsky et al. [20] presented a complete characterization of a dynamic model for a PAM-actuated two-link arm, which can be used for different control designs. The Lagrangian mechanics approach is applied to develop the motion equation with massive links utilizing an experimental approach to approximating the force function, determining the values of certain parameters based on the Adaptive Neural Fuzzy Inference System (ANFIS). The work focused on the model characterization and no control strategies have been addressed.

Enzevaee et al. [21] have presented an Active Force Control (AFC) strategy based on a Fuzzy Logic (FL) controller for tracking control of a single-link robot arm. The simulated and experimental results showed that the proposed control scheme could compensate the subjected disturbances effectively and robustly. The main point which has been reported is that the dynamics of the robot arm were approximated by input–output gain. Moreover, the use of a PID controller as the tracking controller could not compensate the uncertainty in system parameters

Hiroki [22] presented a control method for a two joint leg driven by PAM. The control method is based on adjusting the timing of cyclic input signal using a genetic algorithm (GA) according to simple cost functions. It has been shown that the robustness of the Pam-driven leg is limited by adjustment of input timing. Unfortunately, the work did not support a control design under uncertainties, but rather it has addressed the problem of robustness improvement based on adjustment of cyclic input timing.

Caldwell et al. in [23] presented an indirect adaptive controller based on pole-placement control technique for braided pneumatic muscle actuator (PMA). The model of PAM actuator has been identified via input–output data according to the proposed model structure. The work reported low bandwidth of the closed-loop system and hence low dynamic speed of response due to limited structure of the polynomial model.

The works reviewed in the above literature either used intelligent control schemes, based on fuzzy logic, neural networks, optimization-based control, nonlinear control based on sliding mode control, adaptive backstepping control, or hybrid nonlinear control. Moreover, it is worthy to mention that the structure of the PAM-actuated manipulator differs from one study to another. Different from the previous studies, in this paper, an adaptive controller is developed for tracking control of PAM-manipulator based on synergetic control strategy.

The Synergetic Control (SC) theory is based a state-space theory, which is utilized for the design and control of highly complex and connected nonlinear systems. This control strategy could enable the state variables of the system to evolve on invariant manifolds chosen by the designer and to achieve the required performance in spite of the presence of uncertainties and disturbances [24,25]. The design of nonlinear systems based on synergetic control can follow the following general procedure [26,27]:

1. Forming the extended system of differential equations, which reflects different operations such as achieving the set values, coordinating observing, optimization, suppressing the disturbances etc.
2. Synthesizing “external” controls which ensure a reduction in the extra degrees of freedom of the extended system with respect to the final manifold. The motion of the representing point is described by the equations of the system’s “internal” dynamics.
3. Synthesizing the “internal” controls by means of forming the links between the “internal” coordinates of the system. These links ensure the reaching of the control aim.
4. The synergetic controller directs the trajectories of the system to move onto the manifold from any initial points to their corresponding equilibrium points.

It has been shown that the design parameters of different adaptive and non-adaptive controllers have a direct impact on their performances. These design parameters are often selected based on the trial-and-error procedure. This old technique could not find the optimal dynamic performance of the controlled system based on the proposed controllers. Therefore, a modern optimization technique is used to tune these design parameters to improve the dynamic performance of the controlled system [28,29]. In the present work, the Particle Swarm Optimization (PSO) has been suggested to adjust the design parameters of the controlled system. This modern optimization technique was first proposed by Kennedy in 1995 and it was inspired by the behavior of organisms [30]. This optimization tuner is characterized by fast convergence, the efficiency of computation and it has the capability to find local and global solutions [31,32]. Other modern and generalized optimization techniques can be employed either to improve the optimization process or to make a comparison in performance among each other [33,34,35,36,37,38,39,40,41].

The main problem with systems actuated by Pneumatic Artificial Muscles is that they suffer from external disturbances, uncertainties (e.g., unknown parameters and unmodeled uncertainties, etc.), high nonlinearities, hysteresis and time varying characteristics, which dramatically degrade the performance of tracking control. To handle this important issue, an adaptive control method is proposed for the PAM-actuated one-link robot arm, which can deal effectively with the influence produced by parametric uncertainties in actuating muscles.

The contribution of this paper is to develop an adaptive control algorithm based on synergetic control theory for tracking control of a single-link robot arm actuated by pneumatic artificial muscles under uncertainties in muscle parameters. The proposed controller is continuous and able to prevent chattering and it can simultaneously compensate the parametric uncertainties, and it yields bounded adaptive gains. The improvement in performance of the proposed adaptive controller by PSO-tuning of its design parameters is the second contribution, which can be added as well since it has never been addressed in the literature for such PAM systems. The first controller is based on known parameters of PAM-actuated one-link robot arms named as classical synergetic controller (CSC), while the other adaptive synergetic controller (ASC) solves the problem of unknown uncertainties in system parameters. The stability of the controlled PAM robotic system is analyzed and proved based on Lypunov stability analysis. In addition, the PSO technique is introduced for tuning the designed parameters of proposed controllers to better enhance the performances of the proposed controllers. Thus, the contribution of the present work can be embodied by pursuing the following steps of objectives:

❑ To design a new classical synergetic control (CSC) algorithm for the PAM-actuated robot arm based on Lypunove-based stability analysis.
❑ To design a new adaptive synergetic control algorithm to cope with the problem of uncertainties inherited in parameters of the PAM-actuated robot arm.
❑ To prove the asymptotic stability of the PAM-based robot arm controlled by CSC and ASC, such that all errors finally converge to their corresponding zero equilibrium points based on Lypunove stability analysis.
❑ To better improve the dynamic performance of the PAM-actuated robot arm controlled by proposed controllers by replacing the trial-and-error procedure with the PSO technique for optimal tuning of controllers’ design parameters towards better performance of controllers.

## 2. The Dynamic Model of the PAM-Actuated Single-Link Robot Arm

**Figure 1:**
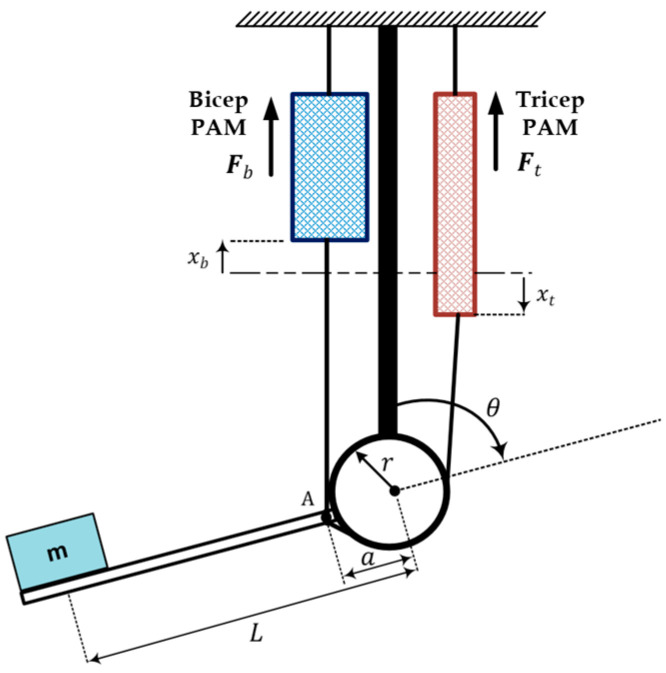
The Pneumatic artificial muscle (PAM) single-link robot arm.

Before proceeding in the control design of the PAM system, it is important to first develop the mathematical model for the system, which mimicks the actual behavior of real system. Then, the system can be analyzed and its associated controller can be designed to meet the required performance. The single link robotic arm is shown in Figure 1 [12].

where, m is the mass (kg), g is the gravitational acceleration ($m/s^{2}$), $B\left(P_{b}\right)$ is the bicep coefficient of viscous friction, L is the length of the arm from the center of the mass to the joint, $B\left(P_{t}\right)$ is the tricep coefficient of viscous friction, $K\left(P_{b}\right)$ represents the bicep spring coefficient (N/m), $K\left(P_{t}\right)$ represents the tricep spring coefficient (N/m). $F\left(P_{b}\right)$ is the force exerted by PAM in the bicep case, $F\left(P_{t}\right)$ is the force exerted by PAM in the tricep case, a is the distance from the joint axis of rotation to the PMs attached point (A), *r* is the pulley radius.

The amount of pneumatic muscle extension $x_{t}$ and muscle contraction $x_{b}$ can be expressed respectively by [12,16],

(1) $$x_{b}=a\;\left(1-cos\;\theta \right)$$

(2) $$x_{t}=a\;\left(1+cos\;\theta \right)$$

The forearm forms an angle $\alpha =sin^{-1}\left(r/a\right)$ with the tricep cable. The forearm is permitted to rotate within this angle. The angle θ=0 corresponds to the case that the forearm is in a downward position, while the angle θ=π represents the case that the forearm is in an extreme upward position. The clockwise torque generated by the bicep muscle on the fore arm is given by:

(3) $$\tau_{cw}=F_{b}\left(.\right)\;a\;sin\theta$$

The counterclockwise torque applied by the tricep muscle is described by:

(4) $$\tau_{ccw}=F_{t}\left(.\right)\;r$$

where $F_{t}\left(.\right)$ and $F_{b}\left(.\right)$ are the developed forces from the tricep and bicep PAMs, respectively, 𝑟 is the pulley radius. The generated $F_{t}\left(.\right)$ and $F_{b}\left(.\right)$ can be described by the following dynamic PAM model [12]:

(5) $$F_{b}\left(.\right)=F\left(P_{b}\right)-K\left(P_{b}\right)x_{b}-B\left(P_{b}\right){\dot{x}}_{b}\;$$

(6) $$F_{t}\left(.\right)=F\left(P_{t}\right)-K\left(P_{t}\right)x_{t}-B\left(P_{t}\right){\dot{x}}_{t}$$

where $F\left(P_{b}\right)$, $K\left(P_{b}\right)\;$and $B\left(P_{b}\right)\;$are the bicep PAM force, spring and viscosity coefficients, respectively, and they are expressed as follows:

(7) $$F\left(P_{b}\right)=F_{0}+F_{1}\;P_{b}\;K\left(P_{b}\right)=K_{0}+K_{1\;}P_{b}\;\;B\left(P_{t}\right)=B_{0t}+B_{1b}P_{b}$$

In addition, $F\left(P_{t}\right)$, $K\left(P_{t}\right)\;$and $B\left(P_{t}\right)\;$represent the tricep PAM force, spring and viscosity coefficients, respectively, and they are defined by the following expressions:

(8) $$F\left(P_{t}\right)=F_{0}+F_{1}\;P_{t}\;K\left(P_{b}\right)=K_{0}+K_{1\;}P_{t}\;\;B\left(P_{t}\right)=B_{0t}+B_{1t}P_{t}$$

It is worthy to note that the coefficient B depends on wether the muscle is being deflated or inflated; that is, one has to differentiate between the tricep and bicep coefficients $B\left(P_{t}\right)$ and $B\left(P_{b}\right)$.

The dynamic equation of motion can be found by summing the torques, described by Equations (3) and (4), about the elbow

(9) $$I\ddot{\theta }=F_{b}\left(.\right)asin\theta -F_{t}\left(.\right)r-MgLsin\theta \;$$

where $I=ML^{2}$ represents the moment of mass inertia about the elbow and the last term (MgLsinθ) has been added to account for the counterclockwise torque exerted on the forearm due to the mass gravity. Substituting Equations (5) and (6) into Equation (9), one can obtain:

(10) $$I\ddot{\theta }=\left(F\left(P_{b}\right)-K\left(P_{b}\right)x_{b}-\left(B_{0b}+B_{1b}P_{b}\right)\;{\dot{x}}_{b}\right)\;a\;sin\theta -\left(F\left(P_{t}\right)-K\left(P_{t}\right)x_{t}-\left(B_{0t}+B_{1t}\;P_{t}\right)\;{\dot{x}}_{t}\right)\;r-MgLsin\theta \;$$

The time derivative of PM extension $x_{t}$ and contraction $x_{b}$ are given, respectively, as:

(11) $${\dot{x}}_{b}=a\;sin\theta .\dot{\theta }\;$$

(12) $${\dot{x}}_{t}=-a\;sin\theta .\dot{\theta }$$

Using Equations (10)–(12), one can get

(13) $$I\ddot{\theta }=\left(F\left(P_{b}\right)-K\left(P_{b}\right)\;x_{b}-a\;\left(B_{0b}+B_{1b}\;P_{b}\right)\;sin\;\theta .\dot{\theta }\right)\;a\;sin\theta -(F\left(P_{t}\right)-K\left(P_{t}\right)x_{t}\;+a\left(B_{0t}+B_{1t}P_{t}\right)sin\theta .\dot{\theta })r-M\;g\;L\;sin\theta \;$$

The pressure of tricep and bicep PAM is given

(14) $$P_{t}=P_{0t}-∆P\;$$

(15) $$P_{b}=P_{0b}+∆P\;$$

where $P_{0t}$,$\;P_{0b}$ are the initial pressure of the tricep and bicep, respectively, Δ𝑃 is assigned as the control input of the system and it describes the pressure difference between the tricep and bicep. Combining Equations (14)–(16), to yield

(16) $$\begin{array}{c} I.\ddot{\theta }=[\left(F_{0}+F_{1}\;P_{0b}\right)+F_{1}\;∆P-a\;\left(K_{0}+K_{1}P_{0b}\right)\;\left(1-cos\;\theta \right)-a\;K_{1}\left(1-cos\;\theta \;\right)\;∆\\ -a\;\left(B_{0b}+B_{1b}\;P_{0b}\right)\;sin\;\theta .\dot{\theta }-a\;B_{1b}\;sin\;\theta .\dot{\theta }\;∆P]a.sin[\;\left(F_{0}+F_{1}\;P_{0t}\right)\\ -F_{1}∆P-a\;\left(K_{0}+K_{1}\;P_{0t}\right)\;\left(1+cos\;\theta \;\right)+a\;K_{1}\;\left(1+\;cos\;\theta \right)\;∆P\\ +a\;\left(B_{0t}+B_{1t}\;P_{0t}\right)\;sin\;\theta .\dot{\theta }-a\;B_{1t}\;sin\;\theta .\dot{\theta }\;∆P]r-M\;gL\;sin\;\theta \end{array}$$

Equation (16) can be rewritten in the following compact form:

(17) $$\ddot{\theta }=f\left(\theta ,\dot{\theta }\right)+b\;\left(\theta ,\dot{\theta }\right)\;∆P\;$$

where

$$f\left(\theta ,\dot{\theta }\right)=\overset{6}{\underset{1}{\sum }}f_{i}\;Z_{i}\left(\theta ,\dot{\theta }\right),\;b\left(\theta ,\dot{\theta }\right)=\overset{6}{\underset{i=1}{\sum }}b_{i}\;Z_{i}\left(\theta ,\dot{\theta }\right)$$

where i=1,2, ⋯,6. The definitions of the elements of the coefficients $f_{i}$, $Z_{i}$ and $b_{i}$ are listed in Table 1.

The variation in the pressure ∆P given in Equation (14) is considered as the control signal; that is u=∆P. In addition, if the state variable $x_{1}$ is assigned to the angular position θ and the state variable $x_{2}$ represents the angular position velocity $\dot{\theta }$, then state space representation can be described by

(18) $$x_{1}=\theta \;{\dot{x}}_{1}=\dot{\theta }=x_{2}\;{\dot{x}}_{2}=\ddot{\theta }=f\left(x_{1},x_{2}\right)+b\left(x_{1},x_{2}\right)\;u$$

## 3. Classical and Adaptive Synergetic Control Design for Single Arm PAM-Actuated Robot

In this section, two control schemes have been developed for tracking control design of angular position for PAM robot arm. The first control design is established based on the classical synergetic control method. The second control design presents the development of an adaptive synergetic control algorithm for tracking control of the PAM robot arm subjected to uncertainties in system parameters.

### 3.1. Synergetic Control Design

Let e be the difference between the actual angle position $x_{1}=\theta$ and the desired trajectory $x_{1d}=\theta_{d}$ as follows:

(19) $$e=x_{1}-x_{1d}\;$$

Using dynamic equation of the PAM robot arm, the first and second derivatives of error equations are given by, respectively,

(20) $$\dot{e}={\dot{x}}_{1}-{\dot{x}}_{1d}=x_{2}-{\dot{x}}_{1d}\;$$

(21) $$\ddot{e}={\dot{x}}_{2}-{\ddot{x}}_{1d}=f+b\;u-{\ddot{x}}_{1d}\;$$

where u is the control signal. For the system described by Equation (18), let the marco-variable ψ(e) be defined as

(22) $$\psi \left(\;e\right)=e+c_{c}\;\dot{e}\;$$

Taking the first-time derivative of Equation (22) gives

(23) $$\dot{\psi }\left(e\right)=\dot{e}+c_{c}\;\ddot{e}\;$$

where $c_{c}$ is a scalar design parameter concerning CSC strategy.

In order to ensure the stability and to guarantee the convergence of the state trajectories to their corresponding desired manifolds and remain on it for future time, the dynamic evolution of the macro-variable towards the manifolds is defined as follows:

(24) $$T\;\dot{\psi }\left(e\right)+\psi \left(e\right)=0\;$$

where T>0 represents the rate of convergence of the macro-variable to manifolds ψ(e)=0.

Using Equations (23) and (24) becomes

(25) $$T\;\left(\dot{e}+c_{c}\;\ddot{e}\right)+\psi \left(e\right)=0\;$$

Using the dynamic equation of the PAM robot arm, Equation (25) becomes

(26) $$T\dot{e}+T\;c_{c}\;\ddot{e}+\psi \left(e\right)=0\;$$

Using Equation (21), one can have

(27) $$T\;\dot{e}+T\;c_{c}\;f+T\;c_{c}\;b\;u-T\;c\;{\ddot{x}}_{1d}+\psi \left(e\right)=0\;$$

In order to ensure the dynamic $T\;\dot{\psi }\left(x\right)+\psi \left(\;x\right)=0$, the control law u can be designed as

(28) $$u=\frac{1}{T\;c_{c}\;b}\;\left(T\;c\;{\ddot{x}}_{1d}-T\;\dot{e}-T\;c_{c}\;f-\psi \left(e\right)\right)\;$$

**Proof.**  The candidate Lypunov function is chosen in terms of micro-variables vector as follows:

(29) $$V=\frac{1}{2}\;\psi^{T}\left(e\right)\;\psi \left(e\right)\;$$

Taking The time derivative of Equation (29) gives

(30) $$\dot{V}=\psi^{T}\left(e\right)\;\dot{\psi }\;\left(e\right)\;$$

Using Equations (24) and (30) becomes

(31) $$\dot{V}=-\frac{1}{T}\;\psi^{2}\left(e\right)\;$$

This indicates that the control law of Equation (28) guarantees the system stability of the PAM robotic System. □

**Theorem** **1.** *For the dynamic equation of the PAM-actuated robot arm, the control signal u can be acquired by Equation (28), which ensures the stability of system motion and stays in the desired manifold.*

### 3.2. Design of Adaptive Synergetic Control for Single-Link Robot Arm

In order to address the problem of uncertainty, which is a characteristic feature in the physical parameters of the PAMs system, and for suppressing the effects of undesired disturbances that may have an effect on the tracking performance, the ASC has been introduced to develop the necessary adaptive laws that can estimate these uncertain parameters such that the asymptotic stability of the adaptive controlled system is guaranteed. In this case, the adaptive syngeneic controller could direct all trajectories of micro-variables to the equilibrium or manifold asymptotically. When the trajectory reaches the manifold, the synergetic controller will maintain it there thereafter.

**Assumption** **1.** *Two coefficients are permitted to be uncertain in their values; namely, the viscous friction coefficient and spring coefficient.*

**Assumption** **2.** *The variation in coefficients of bicep muscle has only been taken into account, that is, the uncertainty in viscosity coefficient* $B_{ob}$ *and the uncertainty in spring coefficient* $K_{ob}$ *. However, since the spring coefficient in the bicep and tricep muscles are the same, then* $K_{ob}$ *is assigned to the coefficient* $K_{o}$ *.*

**Assumption** **3.** *The inflation case will be only considered in developing the control law of the ASC algorithm.*

Referring to Equations (8) and (10), there are three possible parameters of the system, which permit variations in their values; namely, F(p), K(p) and B(p). The assumption 1 indicates that the present work address only the uncertainty in K(p) and B(p), since they are highly subjected to variation during the work of the actuating muscles. According to the above assumptions, the adaptive control law of ASC for the PAM-actuated robot arm will be established based on Lypunov stability analysis. Let ${\hat{B}}_{0b}$ and ${\hat{K}}_{0}$ denote the estimated values of the viscous friction coefficient and spring coefficient, respectively, which are given by

(32) $${\hat{B}}_{0b}=B_{0b}+{\tilde{B}}_{0b}\;$$

(33) $${\hat{K}}_{0}=K_{0}+{\tilde{K}}_{0}\;$$

where, ${\hat{B}}_{0b}$,$\;{\hat{K}}_{0}$ represent the estimated value of $B_{0b}\;\mathrm{and}\;K_{0}$*,* $B_{0b}$ and $K_{0}$ represent the nominal values and ${\tilde{B}}_{0b},\;{\tilde{K}}_{0}$ are the variation in B, K, respectively.

In order to derive the adaptation law, the candidate L.F. has been chosen in terms of estimation errors as follows:

(34) $$V=\frac{1}{2}\;\psi {\left(e\right)}^{2}+\frac{1}{2}\;\gamma_{1}\;{\tilde{B}}_{0b}^{2}+\frac{1}{2}\;\gamma_{2}\;{\tilde{K}}_{0}^{2}\;$$

where, $\gamma_{1},\;\gamma_{2}$ are the adaptation gains. The first time derivative of Equation (34) is given by

(35) $$\dot{V}=\psi \left(e\right)\;\dot{\psi }\left(e\right)+\gamma_{1}\;{\tilde{B}}_{0b}\;{\dot{\hat{B}}}_{0b}+\gamma_{2}\;{\tilde{K}}_{0}\;{\dot{\hat{K}}}_{0}\;$$

Based on Equation (23), one can get

(36) $$\dot{V}=\psi \left(\;x\right)\;\left(\dot{e}+c_{a}\;\ddot{e}\right)+\gamma_{1}\;{\tilde{B}}_{0b}\;{\dot{\hat{B}}}_{0b}+\gamma_{2}\;{\tilde{K}}_{0}\;{\dot{\hat{K}}}_{0}\;$$

where $c_{a}$ is a positive scalar constant concerning the design of the adaptive synergetic controller. Using Equation (21) to have

(37) $$\dot{V}=\psi \left(\;x\right)\;\left[\dot{e}+c_{a}\;f+b\;c_{a}\;u-c_{a}\;{\ddot{x}}_{1d}\right]+\gamma_{1}\;{\tilde{B}}_{0b}\;{\dot{\hat{B}}}_{0b}+\gamma_{2}\;{\tilde{K}}_{0}\;{\dot{\hat{K}}}_{0}\;$$

The ideal control law, given in Equation (28), is no longer applicable in the presence of uncertainty and the actual control law is defined in terms of estimated function$\;\hat{f}$ rather than its ideal one as follows;

(38) $$u=\frac{1}{Tc_{a}\;b}\;\left(-T\;\dot{e}-\psi \left(e\right)-T\;c_{a}\;\hat{f}+T\;c_{a}\;{\ddot{x}}_{1d}\right)\;$$

Substituting Equation (38) in Equation (37) to become

(39) $$\dot{V}=-\frac{1}{T}\;\psi {\left(e\right)}^{2}+c_{a}\;\psi \left(e\right)\;f-c_{a}\;\psi \left(e\right)\;\hat{f}+\gamma_{1}\;{\tilde{B}}_{0b}\;{\dot{\hat{B}}}_{0b}+\gamma_{2}\;{\tilde{K}}_{0}\;{\dot{\hat{K}}}_{0}\;$$

or,

(40) $$\dot{V}=-\frac{1}{T}\;\psi^{2}\left(e\right)+c_{a}\;\psi \left(e\right)\left(f-\hat{f}\right)+\gamma_{1}\;{\tilde{B}}_{0b}\;{\dot{\hat{B}}}_{0b}+\gamma_{2}\;{\tilde{K}}_{0}\;{\dot{\hat{K}}}_{0}\;$$

Since,

$$f=\left(f_{1\;}\;z_{1}+\frac{a^{2}\;K_{0}}{I}\;z_{2}+\frac{a^{2}\;K_{1}P_{ob}}{I}\;z_{2}-\frac{a^{2}\;B_{0b\;}}{I}\;z_{3}-\frac{a^{2}\;B_{1b\;}P_{ob\;}}{I}\;z_{3}+f_{4}\;z_{4}-f_{5}\;z_{5}-f_{6}\;z_{6}\right)$$

and,

$$\hat{f}=\left(f_{1}\;z_{1}+\frac{a^{2}\;{\hat{K}}_{0}}{I}\;z_{2}+\frac{a^{2}\;K_{1}P_{ob}}{I}\;z_{2}-\frac{a^{2}{\hat{B}}_{0b}\;}{I}\;z_{3}-\frac{a^{2}B_{1b\;}P_{ob}}{I}z_{3}+f_{4\;}\;z_{4}-f_{5\;}z_{5}-f_{6\;}z_{6}\right)$$

By subtracting $f-\hat{f}$ and substituting the result in Equation (37), one can have

(41) $$\dot{V}=-\frac{1}{T}\;\psi {\left(e\right)}^{2}+c_{a}\;\psi \left(e\right)\;\left(K_{0}-{\hat{k}}_{0}\right)\left(\frac{\;a^{2}\;z_{2}}{I}\right)-c_{a}\;\psi \left(e\right)\;\left(B_{0b}-{\hat{B}}_{0b}\right)\left(\frac{\;a^{2}\;z_{3\;}}{I}\right)+\gamma_{1}\;{\tilde{B}}_{0b}\;{\dot{\hat{B}}}_{0b}+\gamma_{2}\;{\tilde{K}}_{0}\;{\dot{\hat{K}}}_{0}$$

Since ${\hat{B}}_{0b}=B_{0b}+{\tilde{B}}_{0b}\;$and ${\hat{K}}_{0}=K_{0}+{\tilde{K}}_{0}$, then

(42) $$\dot{V}=-\frac{1}{T}\;\psi {\left(e\right)}^{2}-c_{a}\;\psi \left(e\right)\;{\tilde{K}}_{0}\left(\frac{a^{2}\;z_{2}}{I}\right)+c_{a}\;\psi \left(e\right)\;{\tilde{B}}_{0b}\left(\frac{a^{2}\;z_{3\;}}{I}\right)+\gamma_{1}\;{\tilde{B}}_{0b}\;{\dot{\hat{B}}}_{0b}+\gamma_{2}\;{\tilde{K}}_{0}\;{\dot{\hat{K}}}_{0}\;$$

Equation (42) can be arranged in the form

(43) $$\dot{V}=-\frac{1}{T}\;\psi {\left(e\right)}^{2}-c_{a}\;{\tilde{K}}_{0}\left[\psi \left(e\right)\left(\frac{a^{2}\;z_{2}}{I}\right)-\gamma_{2}\;{\dot{\hat{K}}}_{0}\right]+c_{a}\;{\tilde{B}}_{0b}\left[\psi \left(e\right)\left(\frac{a^{2}\;z_{3}}{I}\right)+\gamma_{1}{\dot{\hat{B}}}_{0b}\right]\;$$

In order to ensure $\dot{V}\leq 0$, the following terms are enforced to be set to zero; that is,

$$\left[c_{a}\;\psi \left(e\right)\left(\frac{a^{2}\;z_{3}}{I}\right)+\gamma_{1}{\dot{\hat{B}}}_{0b}\right]=0\;\left[c_{a}\;\psi \left(e\right)\left(\frac{a^{2}\;z_{2}}{I}\right)-\gamma_{2}\;{\dot{\hat{K}}}_{0}\right]=0$$

Accordingly, the following adaptation laws can be deduced

(44) $${\dot{\hat{B}}}_{0b}=-c_{a}\;\psi \left(e\right)\left(\frac{1}{\;\gamma_{1}I}\right)a^{2}\;z_{3\;}\;$$

(45) $${\dot{\hat{K}}}_{0}=c_{a}\;\psi \left(e\right)\left(\frac{1}{\;\gamma_{2}\;I}\right)a^{2}\;z_{2\;}\;$$

Using Equations (40)–(42) becomes

(46) $$\dot{V}=-\frac{1}{T}\;\psi {\left(e\right)}^{2}\langle 0\;,\;T\rangle 0\;$$

Since the time derivative of L.F ($\dot{V}$) is definitely negative, therefore, the proposed ABSMC can guarantee the asymptotic stability of the system even with the presence of uncertainties in parameters ($B_{0b}$ and $K_{0}$) of the PAM-actuated robot arm.

**Theorem** **2.** *If the dynamic system of the PAM-actuated manipulator is subjected to uncertainty in viscosity coefficient $B_{ob}$ and spring coefficient $K_{ob}$, the developed adaptive laws based on synergetic control methodology described by Equations (44) and (45) could estimate the uncertainties in parameters and ensure the stability of the adaptive controlled robotic system.*

**Figure 2:**
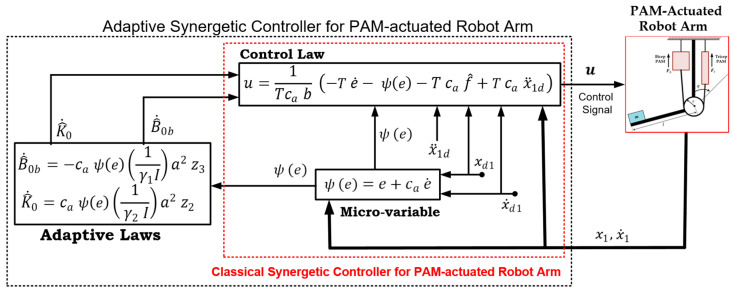
Schematic Diagram of the Proposed Classical and Adaptive Synergetic Controller for Single-link Robot Arm actuated by Pneumatic Artificial Muscles.

Figure 2 shows the schematic diagram of the proposed adaptive synergetic control scheme. The figure also includes the classical synergetic controller enclosed with a red dotted line. However, care has to be taken when assigning some terms inside the control law of both versions of control, where $\hat{f}$ and $c_{a}$ belong to the control law of adaptive control, while f and $c_{c}$ are concerned with control law of the classic synergetic controller.

## 4. Improvement of Controllers’ Performances Based on PSO Technique

In order to improve the performances of the proposed controllers (CMC and ASC), the design parameters of these controllers have to be tuned to towards better performance of the controlled system. Trial-and-error procedure for finding or setting these design parameters is cumbersome and it does not lead to an optimal solution in terms of better dynamic performance of controlled systems. As such, the PSO technique has been suggested to find the optimal values of design parameters. It is well-known that the PSO algorithm has the capability to guide the performance of controllers towards optimality. In the case of CSC, the PSO technique is responsible for tuning the design parameter $c_{c}$, while it undertakes the tuning of constant $c_{a}$ in the case of ASC.

In PSO, each particle navigates around the search (solution) space by updating their velocity according to its own and according to searching experience of other particles. Each particle must update its velocity and position according to the number of iterations, which will be performed according to some cost function to minimize or maximize in each case. In our design, the cost function has to be minimized.

The velocity of each particle is updated according to the following equation [42,43]:

(47) $$V_{i}^{k+1}=w.\;V_{i}^{k}+C_{1}\cdot rand\cdot \left(p_{best}-X_{i}^{k}\right)+C_{2}\cdot rand\cdot \left(g_{best}-X_{i}^{k}\right)\;$$

where, w represents the inertia coefficient, $C_{1}$ represents the personal acceleration coefficient and $C_{2}$ represents the social acceleration coefficient. The position of each particle is updated by the equation [43,44]:

(48) $$X_{i}^{k+1}=X_{i}^{k}+V_{i}^{k+1}\;$$

where $X_{i}^{k}$ and $X_{i}^{k+1}$ represents the current and updated vectors, respectively.

The cost function used to evaluate each particle during the search of the minimum is chosen to be the Root Mean Square Error f (RMSE) function.

The performance of the PSO algorithm is sensitive to PSO parameters. The adjusting of inertia value w strikes a better balance between global exploration and local exploitation. The population size is set to compromise between convergence rate and computation time. Acceleration constants $C_{1}$ and $C_{2}$ represent the particle stochastic acceleration weight toward the personal best ($p_{best}$) and the global best ($g_{best}$). The effect of their setting is either inducing the particle wandering away in the goal area or moving quickly to the goal area. The tuning of PSO parameters can also be performed either using another overlaying optimizer, a concept known as meta-optimization, or even fine-tuned during the optimization. The latter tuning procedure has been adopted in the present work, where the parameters have been tuned for various optimization scenarios as indicated in Table 2.

## 5. Computer Simulation

**Figure 3:**
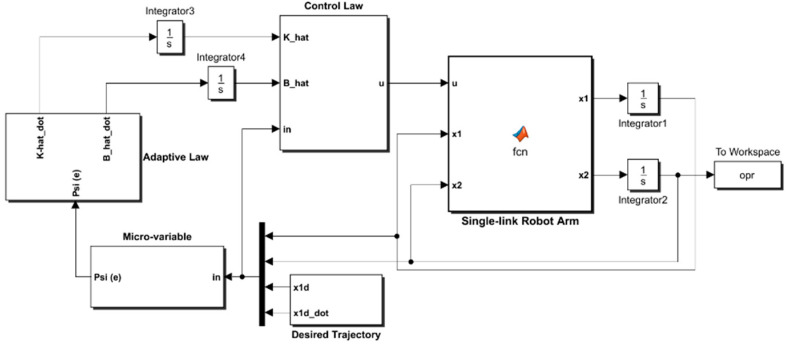
Simulink Model of Adaptive Synergetic Controlled Robotic System.

The numerical values of the pair of PAM-actuated robot arms in the bicep/tricep positions are listed in Table 3. By using the MATLAB/SIMULINK software package, the PAM-actuated robot, based on proposed controllers, has been modeled and coded in Matlab m-functions and Simulink library blocks as shown in Figure 3.

The simulink model of the adaptive synegetic controlled robotic system tries to mimick the block diagram of Figure 2. The model consists of three blocks of matlab functions and two subsystems. The blocks of matlab functions are assigned to the robotic system, adaptive law and control law. The algorithms for control law and adaptive law are written in Matlab code and they are brought to the Simulink environment by the matalb function blocks, which can be drawn by the simulink library. The subsystems used in the simulink model are devoted either to generate trajectory signals or to synthesize the micro-variables of the synergetic controller. The simulation parameters used in the present work include a stop time (60 s), a solver type (ode45) with a variable step based on min step size ($1\times 10^{-5}$ s) and max step size ($1\times 10^{-3}$ s). (One can visit the link cited by Reference [45] for a detailed description of the program source to establish the next scenarios).

**Figure 4:**
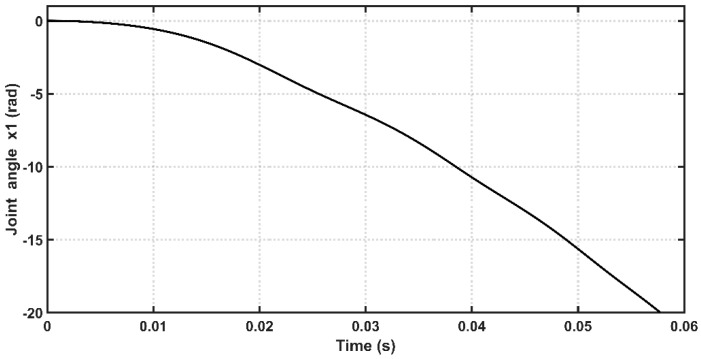
Open loop response for the single-arm robot.

Figure 4 shows the open-loop response for the PAM-actuated single-arm robot tested by sin-wave input of unity amplitude and frequency (1 rad/s).

The design parameters for the CSC are $c_{c}$ and T, while those for the ASC are also$\;c_{a}$ and T. The value of parameter T for CSC is set equal to unity, and that for ASC is assumed to be 0.1. The adaption gain $\gamma_{1}$ in the case of ASC based on bicep coefficient of viscosity $B_{0b}$ has been fixed at value $\gamma_{1}=1\times 10^{-9}$, while adaption gain $\gamma_{2}$ in the case of uncertainty in spring coefficient $K_{0}$ is set with value $\gamma_{2}=1\times 10^{-10}$. In order to reach an improved dynamic performance of the controlled PAM-actuated robot arm, the PSO algorithm has been incorporated to tune the design parameters of CSC and ASC.

**Figure 5:**
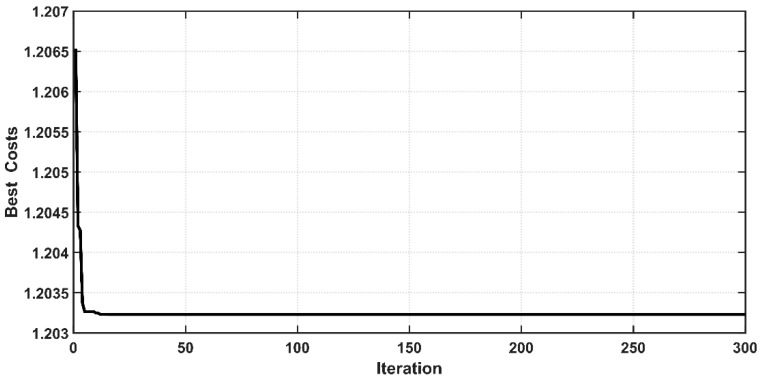
Cost function behavior for classical synergetic control (CSC).

**Figure 6:**
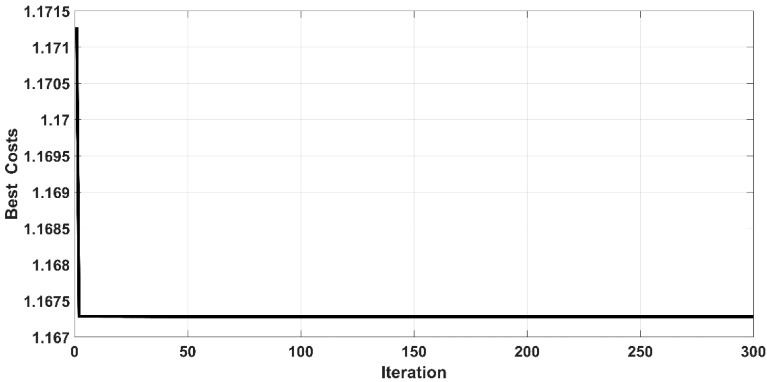
Cost function behavior for adaptive synergetic controller (ASC).

The Root Mean Square Error (RMSE) is the fitness function that has been used to evaluate the iterative particles within the PSO algorithm for all controllers. Figure 5 and Figure 6 show the behavior of cost function with respect to iteration for the controlled PAM single-arm robot based on CSC and ASC, respectively. It is evident that the PSO could successfully guide the design parameters of the proposed controllers to their best solution. However, the PSO algorithm for ASC shows faster convergence than CSC. The convergence of cost function is reached after 15 iterations from the start of the algorithm in the case of CSC, while the cost function finds its global minimum in less than 5 iterations in the case of ASC. The optimal values of tuned parameters will be ready at the end of the algorithm. The controller is termed as “optimal controller” when it is assigned their optimal tuned parameters.

Table 4 gives the set of optimal design parameters based on the PSO technique for the PAM-actuated robot system controlled by CSC and ASC. The other set of design parameters has been chosen on a trial-and-error basis.

Three scenarios are presented in the present work. In the first scenario, the uncertainty is not taken into account, the second scenario considered the uncertainty condition, while the third scenario has been devoted to the validation purpose. The desired angular positions for the first and second scenarios are defined by

$$\theta_{desired}=\frac{\pi }{2}+0.5\;\left(sin\;\left(\;2\;\pi \;f_{1}\;t\right)+sin\left(\;2\;\pi \;f_{2\;}t\right)+sin\left(2\;\pi \;f_{3\;}\;t\right)\right)$$

where, $f_{1}=0.02\;\mathrm{Hz},\;f_{2}=0.05\;\mathrm{Hz}\;\mathrm{and}\;f_{3}=0.09\;\mathrm{Hz}$.

**Scenario I: PAM-actuated Robot Arm based on CSC**

**Figure 7:**
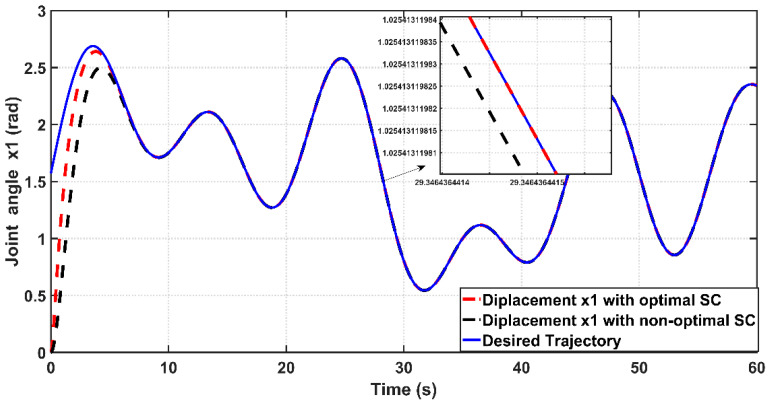
Tracking performance for the PAM actuated robot arm with optimal and non-optimal CSC.

**Figure 8:**
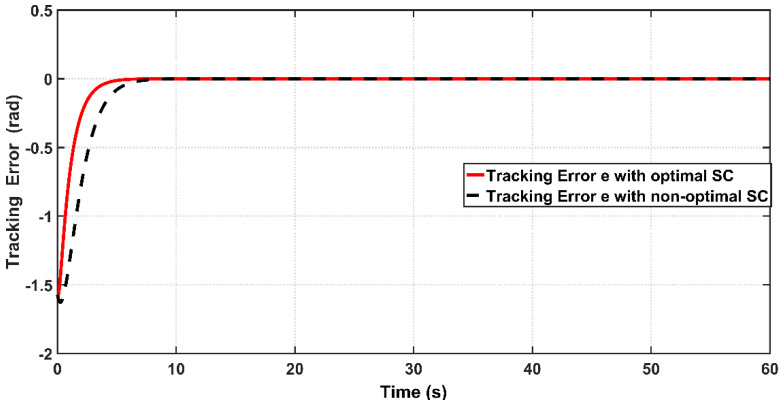
Tracking Error of PAM system controlled by optimal and non-optimal CSCs.

**Figure 9:**
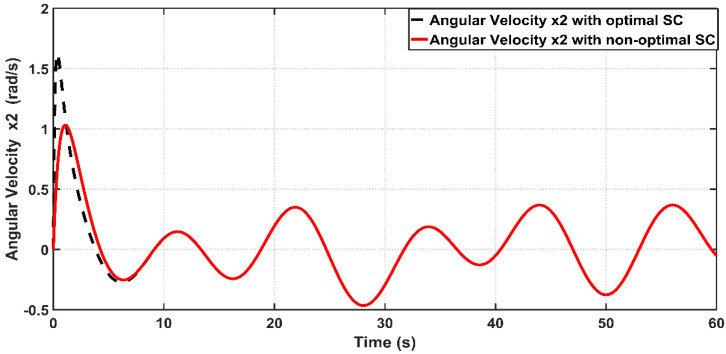
Behaviors of angular velocities for PAM actuated robot arm system controlled with optimal and non-optimal CSC.

Figure 7 shows the tracking performance of the joint angle $x_{1}$ to the desired trajectory based on optimal and non-optimal CSC. According to the Figure 6, the response due to optimal controller shows faster tracking control than that based on non-optimal controller, where the response of optimal CSC reaches its steady-state at 4 s, while the response due to non-optimal CSC reaches its equilibrium at steady state at 6.5 s. The tracking errors (e) for the PAM-actuated robot arm based on optimal and non-optimal CSCs are shown in Figure 8. It is clear from the figure that the optimal CSC gives better performance than the non-optimal version in terms of tracking errors However, to evaluate this one can easily compute the variances of errors resulting from each controller. It has been shown that the RMSE value based on the optimal controller is given by 0.1686 rad, while that based on the non-optimal one is equal to 0.2563 rad. This indicates that the PSO grants the optimal CSC better tracking error performance than its opponent. Figure 9 illustrates the behaviors of angular velocities of the controlled system based on the PSO algorithm and trial-and-error procedure. The velocity of non-optimal controller has higher peak response (1.62 rad/s) than that based on a trial-and-error basis (1.1 rad/s). Therefore, one can conclude that the dynamic response obtained by optimal CSC outperforms that based on a trial-and-error procedure in terms of transient characteristics.

**Figure 10:**
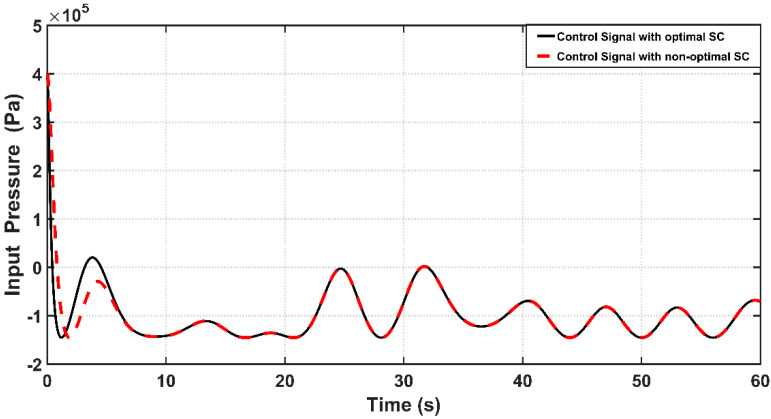
Behavior of control signal u of PAM system controlled by optimal and non-optimal CSC.

The corresponding control effects based on optimal and non-optimal CSC are depicted in Figure 10. One can evaluate the energy or control signal exerted by both controllers by calculating the RMS of the control signal over the entire simulation time. It has been shown that the RMS value of pressure input (control signal) is equal to $\left(1.1367\times 10^{5}\;\mathrm{Pa}\right)$ for the optimal controller, while the value of RMS of the control signal for the non-optimal controller is equal to ($1.1095\times 10^{5}\;\mathrm{Pa}$). This indicates that the optimal controller takes more energy than that based on the non-optimal one. This is the price paid by the optimal controller to have a better dynamic response.

**Scenario II: PAM-actuated Robot Arm Based on ASC**

**Figure 11:**
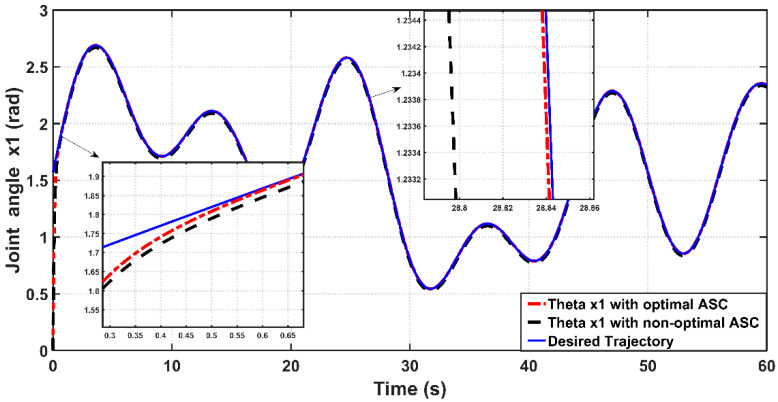
Behaviors of linear angular positions for PAM actuated Robot Arm based on optimal and non-optimal ASC.

In order to design an adaptive controller (ASC), the bicep coefficient of viscosity $B_{0b}$ and the spring coefficient K have been considered as uncertainty factors. The designed ASC will develop adaptive laws, which can estimate the uncertain coefficients ($B_{0b}$,K). The linear angle position behavior based on optimal and non-optimal ASC is shown Figure 11. It is clear from zoomed capture of the figure that the optimal ASC has faster tracking performance than that based on non-optimal ASC. This can be proved numerically, where the response due to the optimal controller concedes with the reverence input after 0.6 s from start-up, while the response based on the non-optimal controller will reach the reference trajectory in 1.3 s.

**Figure 12:**
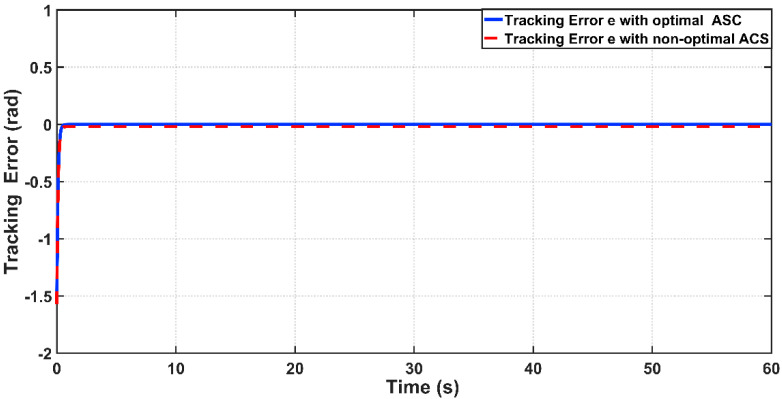
Tracking Error of PAM system controlled by optimal and non-optimal ASC.

**Figure 13:**
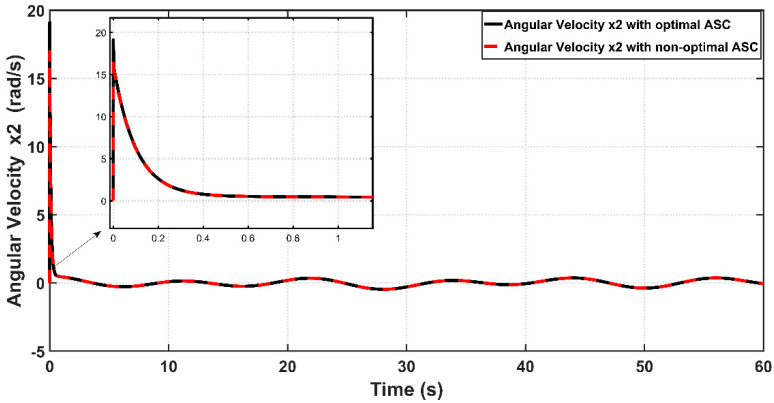
Behavior of joint velocities for optimal and non-optimal ASC.

Figure 12 shows the tracking error (e) between the optimal and non-optimal ASC controlled PAM systems. In a numerical sense, the RMSE value resulting from optimal ASC is equal to 0.0483 rad, while the RMSE given by non-optimal ASC is equal to 0.0530 rad. This indicates that the optimal ASC gives better tracking performance and better error variance than that based on trial-and-error procedure. Figure 13 illustrates the linear velocity behaviors for both optimal and trial-and-error procedure for ASC. However, one can see that the peak of velocity response is slightly higher than that resulting from its counterpart.

**Figure 14:**
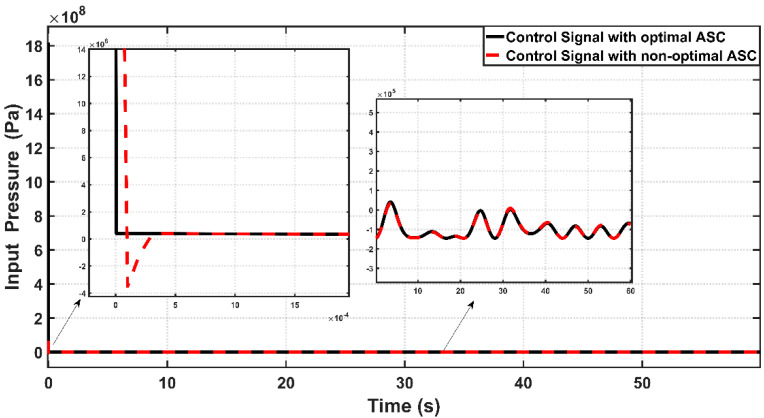
Effects of control signal u based on optimal and non-optimal ASC controlled PAM system.

Figure 14 combines the effects of ASC control signals u due to optimal and trial-and-error procedures. It is interesting to calculate the input pressure variances based on the proposed controllers to evaluate the amount of pressure absorbed by the controlled system. The RMS values of control signals over the entire time of simulation resulting from optimal ASC and non-optimal ASC are$\;7.5494\times 10^{6}\;$and $1.2366\times 10^{6}$, respectively. It is clear that the input pressure taken by optimal ASC is higher than that absorbed by non-optimal controller; this is the price which has to be paid by the optimal controller to have better dynamic and tracking performance.

**Figure 15:**
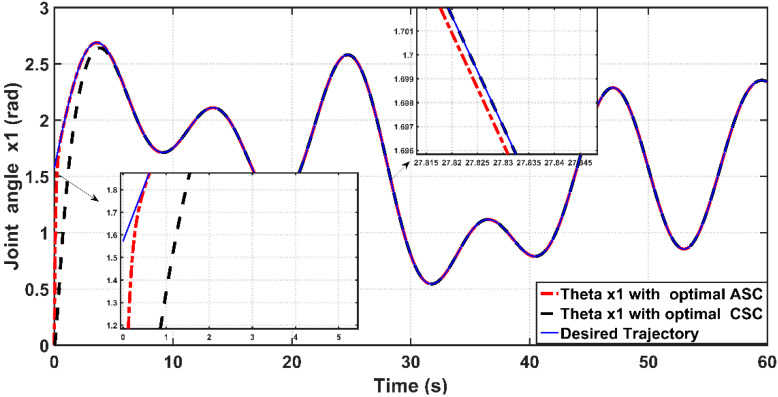
Behaviors of linear angular positions for PAM actuated Robot based on optimal ASC and optimal CSC.

Figure 15 combines the responses based on optimal ASC and optimal CSC. It is clear from the figure that the optimal ASC has less error and hence better tracking performance than optimal CSC. Table 5 and Table 6 reports the numerical values for RMS values of errors and input pressures due to including and excluding the PSO technique. Based on Table 5, one can conclude that the RMSE value in the case of optimal ASC (0.0483 rad) is considerably smaller than that obtained by optimal CSC (0.1686 rad). This indicates the dynamic and tracking performance of optimal ASC outperforms that resulting from optimal CSC. However, this superiority in performance of optimal ASC will be at the price of higher input pressure generated by optimal ASC ($7.5494\times 10^{6}$ Pa) as opposed to lower control signal (input pressure) produced by optimal CSC ($1.1367\times 10^{5}$ Pa).

**Figure 16:**
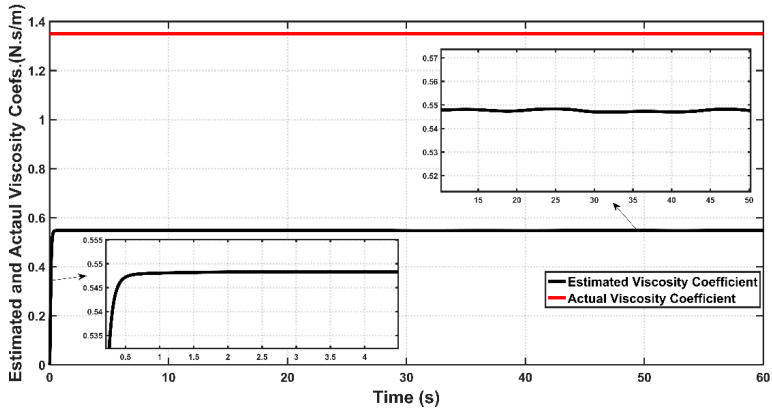
Actual and Estimated Viscosity Coefficients for Bicep muscle of PAM actuated Robot based on ASC.

**Figure 17:**
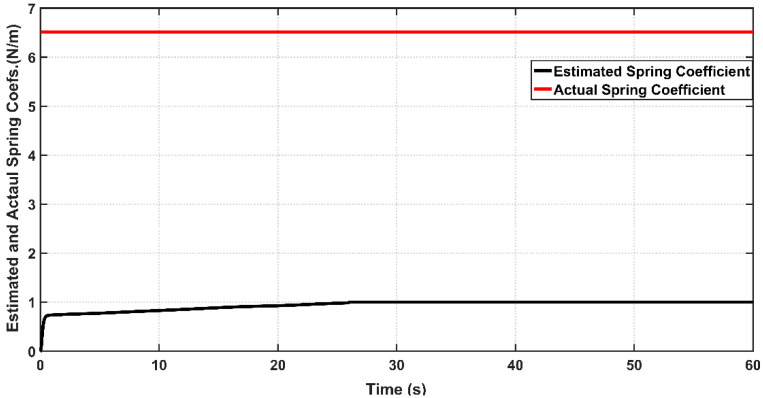
Actual and Estimated Spring Coefficient for Bicep muscle of PAM actuated robot based on ASC.

Figure 16 and Figure 17 show the actual and estimated values of viscosity coefficient $B_{0b\;}$ and spring coefficient K for bicep muscle, respectively. The boundness of the estimation errors of uncertain parameters is one of the essential issues in evaluating the performance of adaptive design for most adaptive controllers. If the adaptive control lacks the ability to confine the estimation errors within certain bounds, then the stability of controlled system may be violated. Based on the figures, one can determine the bounds of$\;{\tilde{B}}_{0b}$ to be ${\tilde{B}}_{0b}\leq 0.8\;$and ${\tilde{K}}_{0}$ to be ${\tilde{K}}_{0}\leq 5.5$ for 0≤t≤60 s. One can conclude that ASC could successfully confine these coefficients within bounded values over the simulation time, and hence the adaptive controller could avoid the instability problem which may arise due to the drift of uncertainty in system parameters.

**Scenario III: Validation of Proposed Controller**

Before proceeding in validation, it is worthwhile to note that the structure of the PAM-actuated single-link robot arm differs from its counterparts with the same link and actuation. In the present work, the cable attached to the pair of muscles that works to rotate the pulley is connected at the end of the robot arm, as shown in Figure 1. Therefore, in order to conduct a fair performance comparison, one has to direct the comparison study with the same robot structure controlled by an adaptive controller. As such, reference [16] has been chosen. However, one problem of the compared work is that it’s proposed adaptive controller has been verified based on a different reference trajectory and different robot system parameters to those used in the present work. Therefore, to be consistent with the compared work, the following desired trajectory is adopted in this scenario:

$$\theta_{d}\left(t\right)=\left[60^{{}^{\circ}}+62.5^{{}^{\circ}}\;\left(sin2\pi \;f_{1}t+0.05sin2\pi \;f_{2}t\right)\right]\;\frac{\pi }{180}$$

where $f_{1}=0.01\;\mathrm{Hz}$ and $f_{2}=0.1\;\mathrm{Hz}$. In addition, the following parameters are used for the PAM-actuated robot arm based on proposed and compared controllers: L=0.5 m, r=0.05/π m, M=50 kg, a=0.025 m. By this setting, the arm is allowed to travel between $\theta =0^{{}^{\circ}}$ (arm fully straightened) to $\theta =180^{{}^{\circ}}$ (arm fully bent). The initial joint angle is set to $42^{{}^{\circ}}$ (0.733 rad) for the controlled system based on both controllers.

**Figure 18:**
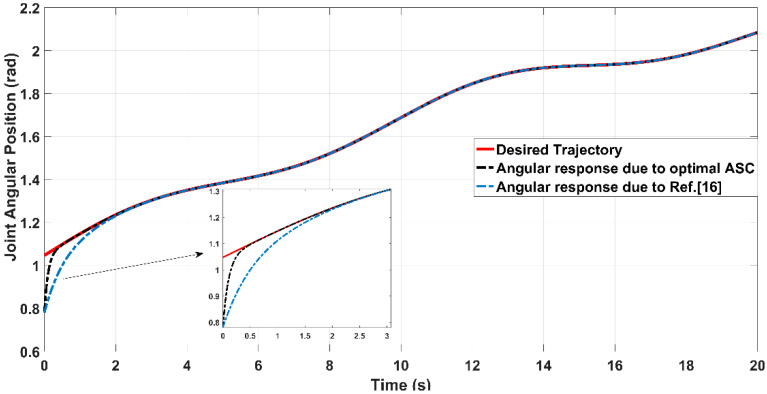
The tracking response based on optimal ASC and the compared controller of reference [16].

Figure 18 shows the tracking performance of the controlled PAM-actuated robot arm based on the optimal adaptive synergetic controller and the compared controller. Starting from the same initial joint condition, it is clear that the joint angular response based on optimal ASC is faster than that based on its counterpart. The angular position resulting from optimal ASC reaches the desired trajectory in 0.65 s, while it coincides with the desired trajectory in 2.5 s in the case of the compared controller. The calculation of RMSE is the other index of evaluation, which is used for comparison. It has been found that the RMSE due to optimal ASC is equal to 0.0147 rad, while this value is equal to 0.0278 rad in the case of the compared controller. This numeric report indicates that the proposed adaptive ASC gives better tracking performance than that given in the previous work.

## 6. Conclusions

This paper presents a new classical and adaptive control design based on a synergetic theory for tracking control of a single-link robot arm actuated by a pair of artificial muscles. The adaptive synergetic controller has been designed to cope with inherited uncertainties in muscle parameters. The design of the proposed controllers has been developed to guarantee the stability of the controlled robotic system.

According to the simulated results, one can conclude that the optimal CSC gives better tracking performance in terms of transient and steady-state characteristics than that obtained by non-optimal CSC. However, the energy drawn by the controlled system based on optimal CSC is higher than that based on the non-optimal controller. In the presence of uncertainty in muscle parameters, the tracking control performance of the adaptive controlled system based on optimal ASC is better than that based on non-optimal ASC. This will be at the expense of higher required input pressure to actuate the muscles in the case of optimal ASC. Moreover, the optimal ASC controller gives better tracking control performance than optimal CSC. Again, this superiority in performance obtained by optimal ASC was on account of higher absorbed energy needed to supply the actuating muscles. Moreover, the optimal ASC could successfully confine the estimation errors of uncertain parameters within certain bounds, which, in turn, will avoid the instability problem due to drifting in estimation errors. Another conclusion that can be drawn is that the proposed adaptive controller generates continuous and monotonic control signals. The proposed controller has been validated with previous work and it has been shown that the proposed controller gives better tracking performance in terms of tracking speed and error than the previous controller.

The future work of the work will focus on real-time implementation and verification of the proposed controller on an actual PAM-actuated robot arm. In addition, a comparison study can be conducted with another adaptive control scheme for the same robot structure, or the PSO technique can be used as a comparison format with other recent optimization techniques.

## Figures and Tables

**Table 1:** Definitions of the elements of coefficients $f_{i}$ , $Z_{i}$ and $b_{i}$.

$Z_{i}$	$f_{i}$	$b_{i}$
$z_{1}=sin\theta$	$f_{1}=\left(aF_{0}+aF_{1}\;P_{ob}-MgL\right)/I$	$b_{1}=a\;F_{1}/I$
$z_{2}=sin\theta \left(cos\theta -1\right)$	$f_{2}=a^{2}\left(K_{o}+K_{1}P_{ob}\right)/I$	$b_{2}=a^{2}\;K_{1}/I$
$z_{3}=sin^{2}\theta .\dot{\theta }$	$f_{3}=-a^{2}\left(B_{0b}+B_{1b}P_{ob}\right)/I$	$b_{3}=-a^{2}B_{1b}/I$
$z_{4}=1+cos\theta$	$f_{4}=a\;r\;\left(K_{o}+K_{1}P_{0t}\right)/I$	$b_{4}=-a\;r\;K_{1}/I$
$z_{5}=sin\theta .\dot{\theta }$	$f_{5}=-a\;r\;\left(B_{0t}+B_{1t}P_{0t}\right)/I$	$b_{5}=a\;rB_{1t}/I$
$z_{6}=1$	$f_{6}=\left(-r\;F_{0}-r\;F_{1}P_{0t}\right)/I$	$b_{6}=r\;F_{1}/I$

**Table 2:** List of parameters assigned to the Particle Swarm Optimization (PSO) algorithm.

Parameters of PSO Technique	Value
The inertia coefficient w	1.4
The personal acceleration coefficient $C_{1}$	2
The social acceleration coefficient $C_{2}$	2
The swarm size (population size)	30
The number of iteration	300

**Table 3:** Numerical values of system parameters.

Coefficient Description	Value
Nominal force exerted by PAM $F_{0}$	$0.986\times 10^{2}\;N$
Variation in force exerted by PAM $F_{1}$	0.803 N
Bicep/nominal viscosity coefficient $B_{0b}$	1.35(N.s/m)
Bicep/variation in viscosity coefficient $B_{1b}$	$4.66\times 10^{-3}\;\left(N.s/m\right)$
Tricep/nominal viscosity coefficient $B_{0t}$	$4.03\times 10^{-1}\;\left(N.s/m\right)$
Tricep/variation in viscosity coefficient $B_{1t}$	$12.0\times 10^{-4}\;\left(N.s/m\right)$
Nominal spring coefficient $k_{0}$	6.51 (N/m)
Variation in spring coefficient $k_{1}$	$2.12\times 10^{-2}\;\left(N/m\right)$
Nominal bicep pressure $P_{ob}$	510.4 kPa
Nominal tricep pressure $P_{ot}$	400 kPa
Mass *M*	20 kg
The distance from mass center to the joint *L*	0.46 m
The distance from PAM attached point to the joint axis a	0.0762 m
Pulley radius *r*	0.0508 m
Gravity Acceleration g	$9.8\;m/s^{2}$

**Table 4:** The optimal setting of design parameters based on the PSO technique.

Controller	Optimal Values		Trial and Error Values	
	Coefficient	Value	Coefficient	Value
CSC	$c_{c}$	0.15	$c_{c}$	1
ASC	$c_{a}$	$3.6533\times 10^{-7}$	$c_{a}$	$1\times 10^{-5}$

**Table 5:** The RMSE values for optimal and non-optimal CSC and ASC.

Controller	PSO	Trial and Error Procedure
CSC	0.1686	0.2563
ASC	0.0483	0.0530

**Table 6:** The RMS value of input pressure for optimal and non-optimal CSC and ASC.

Controller	PSO	Trial and Error Procedure
CSC	$1.1367\times 10^{5}$	$1.1095\times 10^{5}$
ASC	$7.5494\times 10^{6}$	$1.2366\times 10^{6}$
